# Outcome of Concurrent Occult Hemothorax and Pneumothorax in Trauma Patients Who Required Assisted Ventilation

**DOI:** 10.1155/2015/859130

**Published:** 2015-02-16

**Authors:** Ismail Mahmood, Zainab Tawfeek, Ayman El-Menyar, Ahmad Zarour, Ibrahim Afifi, Suresh Kumar, Ruben Peralta, Rifat Latifi, Hassan Al-Thani

**Affiliations:** ^1^Department of Surgery, Section of Trauma Surgery, Hamad General Hospital, P.O. Box 3050, Doha, Qatar; ^2^Department of Emergency, Hamad Medical Corporation, P.O. Box 3050, Doha, Qatar; ^3^Clinical Research, Section of Trauma Surgery, Hamad General Hospital, Doha, Qatar; ^4^Clinical Medicine, Weill Cornell Medical School, P.O. Box 24144, Doha, Qatar; ^5^Internal Medicine, Ahmed Maher Teaching Hospital, Cairo, Egypt

## Abstract

*Background*. The management and outcomes of occult hemopneumothorax in blunt trauma patients who required mechanical ventilation are not well studied. We aimed to study patients with occult hemopneumothorax on mechanical ventilation who could be carefully managed without tube thoracostomy. *Methods*. Chest trauma patients with occult hemopneumothorax who were on mechanical ventilation were prospectively evaluated. The presence of hemopneumothorax was confirmed by CT scanning. Hospital length of stay, complications, and outcome were recorded. *Results*. A total of 56 chest trauma patients with occult hemopneumothorax who were on ventilatory support were included with a mean age of 36 ± 13 years. Hemopneumothorax was managed conservatively in 72% cases and 28% underwent tube thoracostomy as indicated. 29% of patients developed pneumonia, 16% had Acute Respiratory Distress Syndrome (ARDS), and 7% died. Thickness of hemothorax, duration of mechanical ventilation, and development of ARDS were significantly associated with tube thoracostomy in comparison to no-chest tube group. *Conclusions*. The majority of occult hemopneumothorax can be carefully managed without tube thoracostomy in patients who required positive pressure ventilation. Tube thoracotomy could be restricted to those who had evidence of increase in the size of the hemothorax or pneumothorax on follow-up chest radiographs or developed respiratory compromise.

## 1. Introduction

Patients with multiple injuries have a higher incidence of morbidity and mortality if trauma also involves the thorax. Particularly, chest injury is the cause of 10–20% of all trauma related deaths [1–3]. Computed tomography (CT) has high sensitivity for the diagnosis of clinically significant or nonsignificant injuries which provides accurate diagnosis of pleural blood or air in chest trauma patients. Hemopneumothorax (HPTX) is a complex problem following chest injury. The delay in diagnosis and treatment of HPTX is often associated with various complications [4]. Specifically, CT scan can efficiently diagnose HPTX in patients with normal supine chest radiographs which is defined as occult HPTX. Though HPTX is a clinical significant problem, its management is not clearly defined yet [5–7]. Rhea et al. reported 109 chest injuries to be detected by abdominal CT examination, of which 27 had pneumothorax (PTX) and 21 were defined as occult HPTX [6]. Unfortunately, the dimension of occult hemothorax (HTX) or PTX that warrants early drainage (using tube thoracostomy) on initial presentation has not been well established. de Moya et al. [8] proposed an objective scoring system for quantification of occult PTX which could potentially minimize the unnecessary tube thoracostomy for small occult PTX and supported better management decisions. Other investigators have described both qualitative and quantitative evaluation of pleural fluid with upright chest radiographs and pleural sonogram [9, 10].

In the supine position, free pleural fluid collects predominantly in the dependent posterior pleural space and appears as a sickle-shaped lamella on transverse view [9]. This lamella is also visible on chest CT scan and the greatest measured thickness of the lamella could be considered as the reflection of total effusion volume [9, 11].

In earlier practice, patients with PTX who required positive pressure ventilation (PPV) underwent tube thoracostomy to avert progression of tension PTX which might lead to serious life-threatening condition [12]. However, current literature did not clarify the management options for occult PTX particularly in patients who underwent PPV [8]. Therefore, the management of occult PTX is not well defined [12, 13]. Moreover, little is known about the treatment of concurrent occult HPTX in chest trauma patients who are subjected to PPV. Therefore, the present study aims to evaluate the management and outcomes of blunt trauma patients with occult HPTX who need PPV and to determine the role of tube thoracostomy in their management. We hypothesized that occult HPTX in patients with blunt trauma who need PPV or ventilatory support for surgical procedure can be managed conservatively.

## 2. Methods

It is a prospective observational study which included all blunt chest trauma patients who required PPV or ventilatory support for surgical procedure and presented with concurrent HPTX by chest CT (not evident on initial supine chest radiograph) from 2011 through 2013. The presence of HPTX was confirmed by CT evaluation and follow-up chest radiographs were obtained to monitor the progression of HPTX during hospital stay.

Indications for chest tube placement are respiratory compromise with oxygen desaturation and X-ray evidence of pneumothorax or hemothorax progression (increased haziness with obliteration of both costophrenic and cardiophrenic angles).

**Figure 1:**
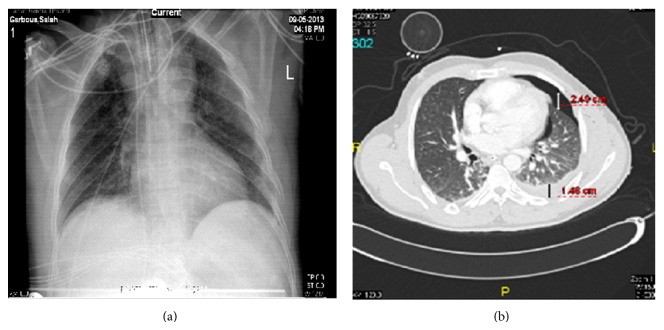
: (a) Chest radiograph and (b) corresponding CT scan of patient with 24 mm pneumothorax and 14 mm hemothorax on left side not identified on chest X-ray.

CT scan was used to quantify PTX by measuring the largest perpendicular distance in millimeters from the chest wall of the largest air pocket. Hemothorax was evaluated by measuring the deepest lamellar fluid stripe at the most dependent portion of the fluid collection (Figure 1). The ventilatory strategy used during the study was synchronized intermittent mandatory ventilation (SIMV) with pressure support mode, tidal volumes of 5 to 7 mL/kg, and controlled mechanical ventilation (CMV) for patient required surgery under general anesthesia. It is noteworthy that ventilator management during the study was not altered by the presence of an occult HPTX.

Data included demographics, mechanism of injury, injury severity score (ISS), chest abbreviated injury scale (AIS), duration of mechanical ventilation, hospital length of stay, and presence of lung contusion. Particular information regarding ventilatory mechanics (such as mode, peak inspiratory pressure, and average tidal volume), indications for tube thoracostomy, complications (pneumonia, empyema, and chest tube complications), and mortality were also recorded. The diagnosis of pneumonia was made on the basis of fever, purulent sputum, infiltrate on chest radiograph, positive culture of endotracheal secretion, and leukocytosis. In addition, all patients with occult HPTX were followed up to determine whether or not a chest tube was placed as part of management and for what indication. CT scans were performed on Siemens Medical Systems, 64-slice scanners scans with the administration of 120 mL of Omnipaque injected at 3 mL/s. Images through the chest were reconstructed at 1.2, 2.5, or 5 mm slice thickness and were analyzed by a consultant trauma radiologist. The Medical Research Center (IRB# 11112/11) at Hamad Medical Corporation, Doha, Qatar, has approved the study.

### 2.1. Statistical Analysis

The data were presented as proportions, medians, or mean ± standard deviation (SD), as appropriate. Patients were divided into two groups according to the need of chest tube insertion or no-chest tube. Differences in categorical variables were analyzed using the chi-square test and the continuous variables were analyzed using Student's* t*-test. For skewed continuous data, nonparametric Mann-Whiteny* U* test was performed. Two tailed *P* values < 0.05 were considered to be significant. Data analysis was carried out using the Statistical Package for the Social Sciences version 18 (SPSS, Inc., Chicago, IL USA).

## 3. Result

**Figure 2:**
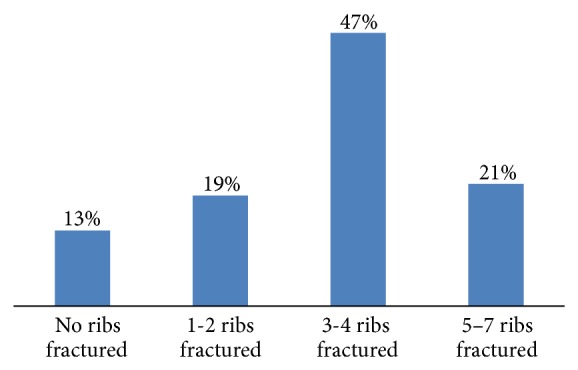
Number of ribs fractured.

**Figure 3:**
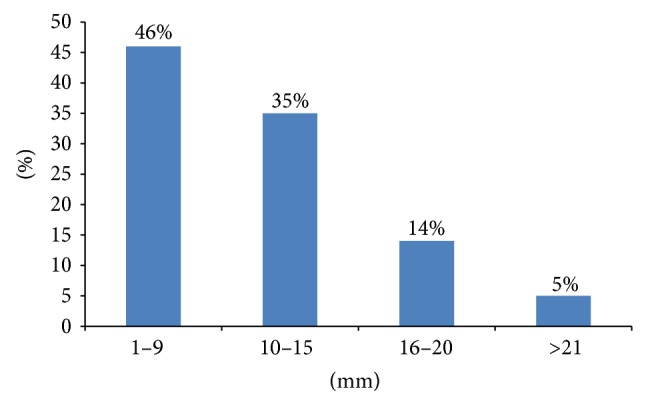
CT scan hemothorax thickness in millimeters.

**Figure 4:**
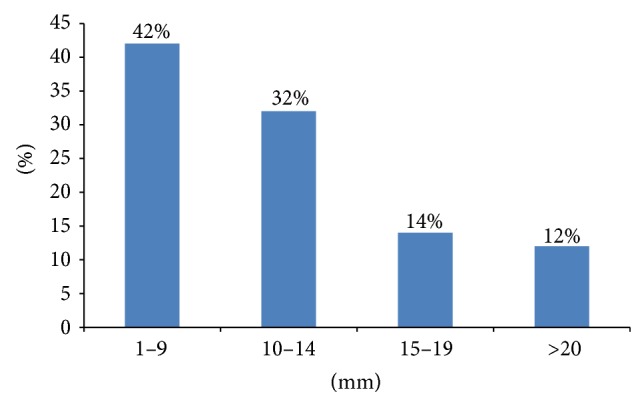
CT scan pneumothorax thickness in millimeters.

A total of 56 chest trauma patients who had occult HPTX and required PPV were prospectively included in the study. The mean age of the patients was 35.8 ± 12.9 years and the majority of cases were males (98.2%). Motor vehicle crash (44.6%) and fall from height (26.8%) were the frequent mechanisms of injury (Table 1). Occult HPTX was successfully managed conservatively in 72% cases and the remaining 28% cases underwent tube thoracostomy as indicated (11 patients had progression of the HTX on follow-up chest radiographs; 2 showed PTX progression, and 2 had increased respiratory distress with oxygen desaturation). Chest tube was placed in 15 patients (13 at ICU and 2 patients needed PPV for surgical intervention within 1–6 days of admission). Lung contusion (84%) represents the most frequent chest injury and 40 (71.4%) patients had multiple rib fracture with a median of 4 (1–7) (Figure 2). On CT evaluation, 46% cases had HTX thickness of ≤9 mm, and 35% had thickness between 10 and 15 mm (Figure 3). The median HTX thickness was 10 mm (range: 1–40). Similarly, larger number of patients (42%) had PTX thickness of ≤9 mm, followed by 10 to 14 mm (32%) and the median PTX thickness was 10.5 mm (range: 2–80) (Figure 4). Seven out of 56 patients had obliteration of costophrenic angle on follow-up chest radiograph but were treated successfully expectantly.

Thirty-seven (66%) patients required intubation and mechanical ventilation due to head injury (GCS below 8) and the remaining 19 (34%) patients required general anesthesia for orthopedic, maxillofacial, or neurosurgical procedures. The ventilator strategy used for these patients was synchronized intermittent mandatory ventilation (SIMV) with pressure support mode, average peak inspiratory pressure of 18.7, and average tidal volumes (505.2 mL) together with controlled mechanical ventilation (CMV). The mean ISS was 24.4 ± 8.7 and chest AIS was 3 ± 2.7. The median mechanical ventilation was 3 (range: 1–21) days and the hospital length of stay was 18 (range: 3–90) days. Ventilator associated pneumonia was observed in 29% cases; 16% had developed ARDS and none of these cases were reported to have empyema. ARDS was reported to occur before tube insertion in the majority.

Table 1 showed a comparison between tube thoracostomy and conservatively treated patients. The two groups were comparable for age, number of fractured ribs, injury severity, presence of pulmonary contusions, and size of PTX. However, duration of mechanical ventilation (6 (1–20) versus 2 (1–21); *P* = 0.02), thickness of HTX (13 (1–40) versus 9 (1–21); *P* = 0.04), and rate of ARDS development (40% versus 7.3%; *P* = 0.003) were significantly higher in patients who underwent tube thoracostomy compared to no-chest tube group. In contrast, patients managed conservatively (no-chest tube) underwent significantly larger number of orthopedic, maxillofacial, or neurosurgical procedures (41.5% versus 13.3%; *P* = 0.04) than tube thoracostomy group. The overall mortality rate was 7% and the two groups were comparable with respect to mortality rate.

## 4. Discussion

To the best of our knowledge this is a unique study that focuses on the management and outcome of occult HPTX in chest trauma patients who need PPV. One of the important findings of our study is that a higher proportion (73%) of chest trauma patients with occult HPTX could be carefully managed conservatively. Our findings are consistent with an earlier prospective multicenter study that demonstrated conservative management in 94% cases of occult PTX [14]. The higher rate of successful observation in that study is due to the fact that they did not consider concurrent HTX and PTX as reported in our series.

Detection of PTX is particularly important for trauma patients who require PPV, as it is associated with the risk of progression to tension PTX and delayed life-threatening manifestations [15–17]. But, in our study, the size of PTX did not correlate with the progression or need for chest tube insertion. However, the size of HTX indicates progression on follow-up and is associated with the need of tube thoracostomy in our patients. Placement of a thoracostomy tube is not without risk and might be involved in incomplete drainage. Moreover, HTX might lead to respiratory distress/failure, retained clot, fibrothorax, empyema, and extended hospitalization [18, 19]. Ball et al. [13] reported major complications in 22% cases that had tube thoracostomy. Similarly, Bailey [20] observed an overall complication rate of 30% related to the procedure but none had insertional complication and only 2% developed major complications (e.g., empyema). Etoch et al. [21] demonstrated that the higher rate of chest tube complications is independently associated with ICU stay and need for mechanical ventilation. So it has been speculated that the selective placement of thoracostomy tubes for such patients might be useful for minimizing the risk of procedural complications.

Interestingly, the CT scan finding for the management of occult PTX remains controversial.

In 1993, Enderson et al. [12] reported the first randomized prospective study on trauma patients with occult PTX to evaluate efficacy of tube thoracostomy. The authors advocated the use of tube thoracostomy in patients with occult PTX who need PPV and also suggested that the size of the initial occult PTX could not predict the progression or formation of a tension PTX. Similarly, other investigators have recommended chest tube insertion in occult PTX patients that required PPV as these patients are at increased risk of developing tension PTX [12, 16, 17].

Over the past decade, using evidence-based practice, the strategies for mechanical ventilation have been significantly improved. An earlier study reported high tidal volumes of 8 to 10 mL/kg, limited positive end-expiratory pressure, and peak inspiratory pressure limits of 40 to 50 cm H_2_O [22]. However, in current practice, decreased airway pressures and tidal volumes are used in the management of critically ill patients. The management guidelines for mechanical ventilation focused on controlling peak and mean airway pressures and recommended relatively much lower pressures than used by earlier investigators [23, 24]. Consistently, lower average peak inspiratory pressure (18.7) and tidal volumes (5 to 7 mL/kg) have been used in our study. Moore et al. [14] suggested that occult PTX is not associated with the progression of respiratory distress and these patients could be safely managed conservatively regardless of the need for PPV.

Traditionally, all posttraumatic HTX cases diagnosed by chest radiograph underwent tube thoracostomy. However, the clinical significance and management of occult HTX has been investigated by several studies and the traditional practice has been changed over time [25–27]. It has been reported that small occult HTX could be managed conservatively in stable patient and the patients with HTX greater than 15 mm on CT are more likely to require drainage. However, limited information is available in the literature to address the management of concurrent occult HTX and PTX. In our series, occult HPTX patients were mainly treated conservatively and only one-third of the cases required tube thoracostomy. We also demonstrated that the size of PTX is not associated with the need of chest tube insertion in patients with PPV. Consistently, an earlier study from our institution reported no correlation between the sizes of PTX and the need for tube thoracostomy in occult HPTX patients [28]. In our series, patients with increased thickness of HTX and those who had ARDS are more likely to have chest tube insertion. Our findings are concordant with earlier studies that identified greater thickness of HTX to be an independent predictor of tube thoracotomy [25–29].

One of the limitations of our study is the small sample size that may affect the power of the study and its findings. However, we attempted to outweigh that by the prospective design. We are, to some extent, confident that our findings are important for the management of occult HPTX patients as other investigators also suggested conservative management of isolated occult PTX [14, 22] and occult HTX [25–27] patients.

In conclusion, occult HPTX can be carefully observed in patients with chest trauma who required positive pressure ventilation. Moreover, delayed tube thoracostomy is not associated with an adverse event. Though occult HPTX is infrequent, awareness of its presence is essential for the emergency physicians and care providers. Close communication with the anesthesiologist is mandatory in patients who require operative intervention. With close observation, the use of tube thoracotomy could be minimized and only restricted to those patients who had evidence of progression of hemo- or pneumothorax (increase in size) on follow-up chest radiographs or developed respiratory compromise. Therefore, the majority of HPTX patients could be spared the potential complications of tube thoracostomy and mechanical ventilation. Further prospective randomized controlled studies are needed to assess larger occult hemothorax thickness in patients who need PPV and to establish clinical guideline based on CT scan volumetric quantification.

## Figures and Tables

**Table 1:** Comparison of patients with chest tube versus no-chest tube.

	Overall	No-chest tube	Chest tube	*P* value
	(*n* = 56)	*n* = 41 (%)	*n* = 15 (%)	
Age (mean ± SD)	35.8 ± 12.9	36 ± 12.7	35.3 ± 14.1	0.85
Males *n*. (%)	55 (98.2)	41 (100%)	14 (93.3)	0.09
Mechanism of injury *n*. (%)				0.13
Motor vehicle crash	25 (44.6)	20 (48.8)	5 (33.3)	
Fall from height	16 (26.8)	13 (31.7)	3 (20)	
Pedestrian	10 (17.9)	6 (14.6)	4 (26.7)	
Stab	1 (1.8)	1 (2.4)	0 (0)	
Other	4 (7.1)	1 (2.4)	3 (20)	
Lung contusion *n*. (%)	47 (83.9)	33 (80.5)	14 (93.3)	0.25
Number of fractured ribs (median; range)	4 (1–7)	4 (1–6)	4 (1–7)	0.59
Hemothorax thickness (median; range)	10 (1–40)	9 (1–21)	13 (1–40)	0.04
Pneumothorax thickness (median; range)	10.5 (2–80)	10 (2–70)	12 (2–80)	0.12
Injury severity score (mean ± SD)	24.4 ± 8.7	24.3 ± 9.5	24.9 ± 6.5	0.79
Chest AIS (mean ± SD)	3 ± 2.7	2.98 ± 0.27	2.93 ± 0.25	0.60
Surgical procedures^*^ *n*. (%)	19 (33.9)	17 (41.5)	2 (13.3)	0.04
Ventilatory days (median; range)	3 (1–21)	2 (1–21)	6 (1–20)	0.02
Hospital length of stay (median; range)	18 (3–90)	17 (3–90)	18 (5–90)	0.42
Ventilator-associated pneumonia *n*. (%)	16 (28.6)	12 (29.3)	4 (26.7)	0.84
Acute Respiratory Distress Syndrome *n*. (%)	9 (16.1)	3 (7.3)	6 (40)	0.003
Mortality *n*. (%)	4 (7.1)	2 (4.9)	2 (13.3)	0.28

^*^Orthopedic, maxillofacial, or neurosurgery.
